# Nuclear BAG-1 expression reflects malignant potential in colorectal carcinomas

**DOI:** 10.1038/sj.bjc.6600579

**Published:** 2002-11-04

**Authors:** R Kikuchi, T Noguchi, S Takeno, Y Funada, H Moriyama, Y Uchida

**Affiliations:** Department of Surgery II, Oita Medical University, 1-1 Idaigaoka, Hasama-machi, Oita 879-5593, Japan

**Keywords:** BAG-1, colorectal cancer, immunohistochemistry, prognosis

## Abstract

BAG-1 is a recently identified Bcl-2-interacting anti-apoptotic protein. The aim of our study was to investigate the immunohistochemical staining pattern of BAG-1 protein in patients with colorectal cancer and examine associations of BAG-1 expression with various clinicopathological factors and patient survival. Tumour samples were collected from 86 patients diagnosed with colorectal cancer. There was significant variation in the immunohistochemical staining patterns for BAG-1, including absent staining and staining of either the cytoplasm, nucleus or both. Twenty-one colorectal carcinomas (24.4%) exhibited a nuclear staining pattern whilst 56 (65.1%) exhibited cytoplasmic staining. The percentage of cases exhibiting nuclear BAG-1 positivity was significantly higher in distant metastasis-positive cases (55.6%) than in distant metastasis-negative cases (20.8%; *P*=0.036). Overall survival was significantly shorter for patients with tumours exhibiting BAG-1 positive nuclei than those with absent nuclear BAG-1-staining (*P*=0.011). In addition, the multivariate cox proportional hazard models indicated that nuclear BAG-1 expression was the only independent prognostic variable for mortality (*P*=0.013). These studies demonstrate that nuclear BAG-1 expression is a useful predictive factor for distant metastasis and a poor prognosis in patients with colorectal cancer.

*British Journal of Cancer* (2002) **87**, 1136–1139. doi:10.1038/sj.bjc.6600579 www.bjcancer.com

© 2002 Cancer Research UK

## 

Recent studies have suggested that apoptosis is controlled by a variety of genes, with dysregulation of these genes playing an important role in the pathogenesis of many human diseases including cancer (Oltvai *et al*, 1993; Soini *et al*, 1996; Koshida *et al*, 1997). BAG-1 protein was originally identified as a novel regulator of apoptosis by virtue of its ability to bind Bcl-2, a potent inhibitor of cell death, and it not only independently inhibits apoptosis but also enhances the anti-apoptotic activity of Bcl-2 (Takayama *et al*, 1995). Moreover, variations in BAG-1 levels parallel alterations in cellular proliferation and viability (Clevenger *et al*, 1997). Despite extensive studies of the function of BAG-1, the exact role of BAG-1 in the carcinogenesis and progression of human colorectal cancers remains unclear. In addition, discrepant results regarding BAG-1 immunostaining and patient survival in early breast cancer have been in dispute (Tang *et al*, 1999; Turner *et al*, 2001). In the present study, we used a polyclonal antibody that allowed specific detection of human BAG-1 protein and examined the immunohistochemical staining patterns of BAG-1 protein in patients with colorectal cancer. We also determined associations between the BAG-1 expression pattern and various clinicopathological factors and patient survival.

## MATERIALS AND METHODS

### Patients and tumour samples

A total of 86 adenocarcinomas of the colon and rectum were studied. Tumours were obtained surgically between 1991 and 1995 at the Department of Surgery II, Oita Medical University. All specimens were fixed in 10% buffered formalin and embedded in paraffin.

### Immunohistochemical staining

Deparaffinized and rehydrated specimens were heated in 10 mM citrate buffer, pH 6.0, for 10 min in an autoclave at 121°C. After treatment with 10% normal goat serum for 10 min to block nonspecific protein binding, polyclonal BAG-1 antibody (rabbit antimouse, clone C-16; Santa Cruz CA, USA; 250 X dilution) was applied. Tissue sections were incubated overnight at 4°C. After brief rinsing, the Catalyzed Signal Amplification system (DAKO Corp.) was used according to the manufacturer's instructions to visualise specific BAG-1 staining. After brief washing, sections were incubated with diaminobenzidine and H_2_O_2_ for 5 min. Sections were then lightly counterstained with haematoxylin, dehydrated in graded alcohols, cleared in xylene and coverslipped.

The immunopositive cell area was used for evaluation of the immunohistochemical staining of BAG-1 antibody: negative 0–10%; positive >10%. Expression of BAG-1 was also evaluated in terms of immunostaining of the tumour cell nucleus and cytoplasm. A clinicopathological study was performed by reference to mean tumour diameter, depth of invasion, histological grade of adenocarcinoma and the presence of lymph node or distant metastasis.

### Statistical analysis

Correlations between antigen expression and the various clinicopathological factors (mean tumour diameter, depth of invasion, histological grade of adenocarcinoma and the presence of lymph node or distant metastasis) were examined by the Student's *t*-test, chi-squared test, Fisher's exact probability or Mann–Whitney's *U*-test. Overall survival was calculated according to the Kaplan–Meier method, from the time of operation to either death or date of last follow-up, and the log-rank test was used to determine statistical differences between life tables. The multivariate cox proportional hazards model was used to determine whether any of the factors tested (tumour diameter, depth of invasion, histological grade of adenocarcinoma, lymph node metastasis and nuclear or cytoplasmic immunohistochemical expression for BAG-1) could be identified as independent prognostic factors for overall patient survival. A *P* value of less than 0.05 was considered statistically significant.

## RESULTS

### Immunohistochemical staining of BAG-1

**Figure 1:**
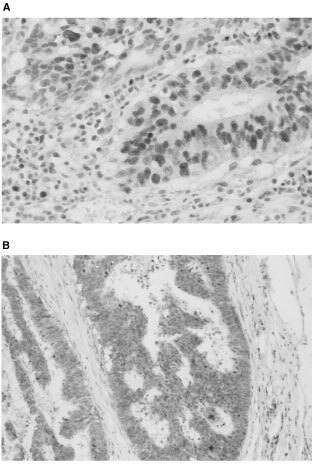
Immunolocalisation of BAG-1 in samples of human colorectal carcinomas. Immunostaining revealed BAG-1 is immunostained in the tumour cell nucleus (**A**) and the cytoplasm (**B**: Original magnification ×400).

There was significant variation in the immunohistochemical staining patterns of BAG-1 with tumours exhibiting absent staining and staining of either the cytoplasm, nucleus or both. The distinction between cytoplasmic and nuclear BAG-1 staining is demonstrated in Figure 1A,B

. Twenty-one colorectal carcinomas (24.4%) exhibited a nuclear staining pattern whilst 56 (65.1%) exhibited a cytoplasmic staining pattern.

### Correlations between the expression of BAG-1 and the various clinicopathological factors

Table 1

**Table 1:**
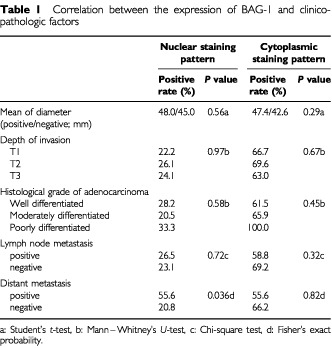
Correlation between the expression of BAG-1 and clinicopathologic factors

shows the correlations between the expression of BAG-1 and various clinicopathological factors. The percentage of tumours exhibiting nuclear BAG-1 positivity was significantly higher in cases positive for distant metastases (55.6%) compared to cases without distant metastases (20.8%; *P*=0.036; Fisher's exact probability). No other significant correlation was evident between nuclear or cytoplasmic BAG-1 expression and the various clinicopathological factors analysed.

### Survival analysis

**Figure 2:**
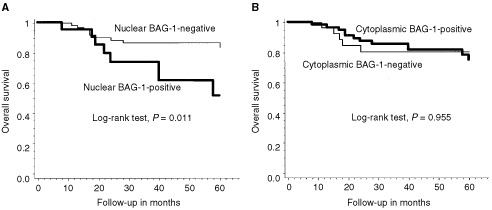
Overall survival probability in relation to nuclear (**A**) and cytoplasm (**B**) BAG-1 expression status.

Overall survival, as determined by the Kaplan–Meier analysis, was significantly shorter for patients with tumours exhibiting nuclear BAG-1 positivity compared to those that were negative for nuclear BAG-1 staining (Figure 2A

; log-rank test, *P*=0.011). Survival at 5 years was 51.3% in patients with tumours exhibiting nuclear BAG-1 positivity compared to 83.9% in those patients with tumours that were negative for nuclear BAG-1 staining. There was no significant difference in survival between patients with cytoplasmic BAG-1-positive tumours and those with cytoplasmic BAG-1-negative tumours (Figure 2B; log-rank test, *P*=0.955). Using the variables of tumour diameter, depth of invasion, histological grade of adenocarcinoma, lymph node metastasis, nuclear BAG-1 expression and cytoplasmic BAG-1 expression, the multivariate cox proportional hazard model indicated that nuclear BAG-1 expression was the only independent prognostic variable for mortality (Table 2

**Table 2:**
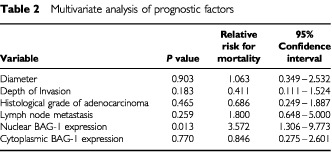
Multivariate analysis of prognostic factors

: hazard ratio=3.572, *P*=0.013). The other clinicopathological factors were not significantly associated with patient survival.

## DISCUSSION

There have been few reports regarding the immunohistochemical staining pattern of BAG-1 in colorectal cancer. Immunohistochemical expression of BAG-1 detectable by antibody (C-16) staining is weak in colorectal mucosa (Takayama *et al*, 1998). Use of the standard avidin–biotin–peroxidase complex (ABC) technique that we usually employ (Kikuchi *et al*, 2000) indicates that approximately 5% of colorectal cancer cells exhibit nuclear or cytoplasmic positivity (data not shown). Hence, we used the Catalyzed Signal Amplification system (DAKO Corp.), which is an extremely sensitive immunocytochemical visualisation system to facilitate the accurate determination of the expression of BAG-1 in colorectal cancers. Indeed, this is the first large-scale retrospective study to assess the prognostic significance of BAG-1 expression in patients with colorectal cancer. We noted that tumour cells exhibited two patterns of immunohistochemical staining *viz* cytoplasmic or nuclear. Comparable cytoplasmic or nuclear staining patterns have been reported in other cancers (Brimmell *et al*, 1999; Tang *et al*, 1999; Yamauchi *et al*, 2001). The reason is as follows: the bag-1 gene of humans and mice can produce two major proteins as a result of alternative translation initiation sites in a common mRNA. The shorter isoform (BAG-1) is predominantly a cytoplasmic protein, while the longer isoform (BAG-1L) is mostly translocated to the nucleus through its nuclear localisation signal (Packham *et al*, 1997; Takayama *et al*, 1998). The BAG-1 antibody (C-16) used in this study should recognise all isoforms (Crocoll *et al*, 2000). In addition, the intracellular localisation of BAG-1 may be modulated by cellular conditions or the differentiation status of these epithelial cells (Yamauchi *et al*, 2001). As a result, the BAG-1 protein may be immunolocalised to the cytoplasm or nucleus in colorectal cancer cells.

There are several reports to indicate that the expression of BAG-1 correlates with the malignant potential of other carcinomas (Tang *et al*, 1999; Shindoh *et al*, 2000). We then studied the relationship between BAG-1 expression and clinicopathological factors and prognosis. The nuclear expression of BAG-1 correlated with the presence of distant metastases. In addition, the prognosis of patients with nuclear BAG-1-positive tumours was significantly worse than that of those with nuclear BAG-1-negative tumours. In contrast, the cytoplasmic expression of BAG-1 was not related to the clinicopathological factors examined or patient prognosis. Therefore, the nuclear expression of BAG-1 was impressively correlated with the malignant potential in colorectal cancer. BAG-1 has been reported to facilitate epithelial cell survival following detachment from the underlying extracellular matrix (Ruoslahti, 1996; Weaver *et al*, 1996) and to promote cell migration in human gastric cancer cells (Naishiro *et al*, 1999). These functions could contribute to the development of distant metastases in malignant tumours since the overexpression of BAG-1 in melanoma cells increases the metastatic potential of these tumour cells (Takaoka *et al*, 1997). In our study, the percentage of cases exhibiting nuclear BAG-1 positivity was significantly higher in distant metastasis-positive cases than in distant metastasis-negative cases. Previous studies have reported that the gain-of-function p53 mutants derive from human tumours upregulated the transcription of BAG-1 RNA and the expression of a reporter gene from the BAG-1 promoter (Yang *et al*, 1999). These data are very interesting, since the function of BAG-1 may be associated with carcinogenesis or malignant potential acting through mutant-p53 functions. In summary, we can conclude that nuclear BAG-1 expression is an indicator of malignant potential and is a poor prognostic marker in colorectal carcinoma.

Finally, we discuss the significance of the nuclear BAG-1 expression. The shorter BAG-1 isoform is predominantly a cytoplasmic protein, while the longer isoform (BAG-1L) is mostly translocated to the nucleus (Packham *et al*, 1997; Takayama *et al*, 1998). Moreover, BAG-1L protein is rarely expressed in normal tissues but is commonly expressed by tumour cell lines, and a change in BAG-1 mRNA translation frequently accompanies malignant transformation (Takayama *et al*, 1998). In short, the nuclear BAG-1 expression almost certainly indicates BAG-1L, and is likely to be relevant to the malignant potential. Interestingly, several studies have reported that nuclear BAG-1 expression correlated with reduced survival in patients with invasive breast cancer (Tang *et al*, 1999) and laryngeal cancer after radiation therapy (Yamauchi *et al*, 2001). Thus, it is reasonable to suppose that nuclear BAG-1 expression is closely related to the malignant potential. However, two papers reported that cytoplasmic BAG-1 expression represented a potential marker of improved survival in early-stage breast cancer patients (Turner *et al*, 2001) and nonsmall cell lung cancer patients (Rorke *et al*, 2001) whilst nuclear BAG-1 expression was not related to the malignant potential. It is likely that the discrepancy between results may be attributed to different antibody or organ specificity. We believe that it is not clear whether the discrepant results are in conflict, since the differences of BAG-1 localisation in cancer cells indicate different expression of BAG-1 isoforms, which may well have different biological functions. Our data strongly indicate that further studies are necessary in order to clarify the relationship between BAG-1 isoforms and BAG-1 functions.
